# Exploring resilience models in a sample of combat-exposed military service members and veterans: a comparison and commentary

**DOI:** 10.1080/20008198.2018.1486121

**Published:** 2018-07-02

**Authors:** Christina M. Sheerin, Kelcey J. Stratton, Ananda B. Amstadter, The VA Mid-Atlantic Mental Illness Research, Education, Clinical Center (MIRECC) Workgroup, Scott D. McDonald

**Affiliations:** a Psychology Service, Hunter Holmes McGuire VA Medical Center, Richmond, VA, USA; b Virginia Institute for Psychiatric and Behavioral Genetics, Virginia Commonwealth University, Richmond, VA, USA; c Department of Psychology, Virginia Commonwealth University, Richmond, VA, USA; d VA Mid-Atlantic Mental Illness Research, Education and Clinical Center, Durham VA Medical Center, Durham, NC, USA; e Department of Physical Medicine and Rehabilitation, Virginia Commonwealth University, Richmond, VA, USA

**Keywords:** Resilience, psychological, veterans, stress disorders, post-traumatic, military combat exposure, Resiliencia, psicológico, veteranos, trastornos de estrés, Postraumático, exposición a combate militar, 韧性，心理, 退伍军人, 应激障碍，创伤后, 军事战争暴露, • To illustrate the impact of various operational definitions on our understanding of resilience, we compared four definitions in the same sample of military personnel exposed to combat trauma (*N* = 849).• The results empirically demonstrate that resilience classification varies across definitions and in this combat-trauma exposed sample, many of whom were treatment-seeking, many categorized as resilient by various definitions also had current psychiatric conditions.• Examination of multiple domains of resilience will shed new light on our understanding of this construct.

## Abstract

**Background**: The term resilience is applied in numerous ways in the mental health field, leading to different perspectives of what constitutes a resilient response and disparate findings regarding its prevalence following trauma.

**Objective**: illustrate the impact of various definitions on our understanding and prevalence of resilience, we compared various resilience definitions (absence of PTSD, absence of current mental health diagnosis, absence of generalized psychological distress, and an alternative trauma load–resilience discrepancy model of the difference between actual and predicted distress given lifetime trauma exposure) within a combat-exposed military personnel and veteran sample.

**Method**: In this combat-trauma exposed sample (*N* = 849), of which approximately half were treatment seeking, rates of resilience were determined across all models, the kappa statistic was used to determine the concordance and strength of association across models, and *t*-tests examined the models in relation to a self-reported resilience measure.

**Results**: Prevalence rates were 43.7%, 30.7%, 87.4%, and 50.1% in each of the four models. Concordance analyses identified 25.7% (*n* = 218) considered resilient by all four models (kappa = .40, *p* < .001). Correlations between models and self-reported resilience were strong, but did not fully overlap.

**Conclusions**:The discussion highlights theoretical considerations regarding the impact of various definitions and methodologies on resilience classifications, links current findings to a systems-based perspective, and ends with suggestions for future research approaches on resilience.

## Abstract (es)

**Antecedentes**: El término resiliencia se ha aplicado de muchas maneras en el campo de la salud mental, llevando a diferentes perspectivas de lo que constituye una respuesta resiliente y hallazgos dispares relacionados a la prevalencia después de un trauma.

**Objetivos**: Para Ilustrar el impacto de las diversas definiciones en nuestra compresión y prevalencia de la resiliencia, comparamos varias definiciones de resiliencia (ausencia de TEPT, ausencia de un diagnóstico de salud mental actual, ausencia de malestar psicológico generalizado, y un modelo alternativo de discrepancia entre la carga y la resiliencia del trauma de la diferencia entre la angustia real y la prevista dada la exposición al trauma a lo largo de la vida) entre personal militar expuesto a combate y una muestra de veteranos.

**Método**: En esta muestra expuesta a trauma de combate (*N*= 849), de la cual aproximadamente la mitad estaba en búsqueda de tratamiento, las tasas de resiliencia se determinaron en todos los modelos, el kappa estadístico se usó para determinar la concordancia y la fuerza de asociación en todos los modelos, y el *t*-test examinó los modelos en relación a la medida de resiliencia auto-reportada.

**Resultados**: La tasas de prevalencia variaron desde 43,7%, 30,7%, 87,4% y 50,1% en los cuatro modelos respectivamente. El análisis de concordancia identificó un 25,7% (*n*=218) considerado resiliente por los cuatro modelos (kappa= .40, *p*< .001). Las correlaciones entre los modelos y la resiliencia auto-reportada fueron fuertes, pero no se superpusieron por completo.

**Conclusiones**: la discusión resalta las consideraciones teóricas sobre el impacto de las distintas definiciones y metodologías sobre las clasificaciones de resiliencia, vincula el hallazgo actual con una perspectiva basada en sistemas y finaliza con sugerencias para futuros enfoques de investigación en resiliencia.

## Abstract (zh)

**背景**：韧性这个术语在心理健康领域有多种使用方式，导致对于韧性反应的组成有不同观点，以及对其在创伤后发生率有不同结论。

**目的**：为了说明各种韧性的定义对我们理解及其发生率的影响，我们在战争暴露后的兵役成员和退伍军人样本中对不同的模型进行了比较，包括：没有创伤后应激障碍，没有当前的心理健康诊断，没有一般的心理困扰，以及一种在特定终生创伤暴露前提下关于实际和预测痛苦的创伤负荷—韧性差异模型。

**方法**：在这个创伤暴露样本中（*N* = 849），其中大约一半来自寻求治疗人员，在所有模型中计算了韧性的发生率，使用kappa 系数确定不同模型之间关联的一致性和强度，*t*-test 考察了与一个自评韧性测量模型的关系。

**结果**：四种模型的患病率分别为43.7％，30.7％，87.4％和50.1％。 一致性分析发现，样本的25.7％（*n* = 218）在全部四个模型均认为有弹性（kappa = .40，*p* <.001）。 模型与自我报告的韧性之间的相关性很强，但没有完全重叠。

**结论**：讨论强调了理论考虑上多重定义和方法对韧性分类的影响的因素，将当前的研究结果与一个基于系统的观点联系起来，最后提出针对韧性未来研究方法的建议。

## 1. Introduction

Conceptualizations of resilience imply a relative resistance to distress and disturbance from adverse life experiences, or positive adaptation despite serious environmental challenges and risks (Luthar, Cicchetti, & Becker, 2000; Masten, Best, & Garmezy, 1990). There has been an increasing interest in resilience in recent years and growing efforts have been made to increase resilience, particularly in populations where stressor exposure is expected, such as military samples (Casey, 2011; Reivich, Seligman, & McBride, 2011). Resilience, however, represents a multifaceted and multideterminant (i.e. made up of a variety of factors) construct; as such, the concept is difficult to measure and the term resilience has been applied in numerous ways in the literature. Thus, the existing research findings are disparate, with widely differing estimates of the prevalence of this phenotype in the post-trauma context (see Bonanno, 2004; Luthar et al., 2000, for review), leading to difficulties comparing resilience across studies.

Prevalence rates of resilience across different samples in the literature vary, probably in part owing to differing definitions of this construct; for example, Infurna and Luthar (2016a) demonstrated differences in prevalence based on various approaches to modelling resilience outcomes. Among the various definitions, resilience often refers to traits or capacities, including self-reported responding and coping (Blackburn & Owen, 2016; Vyas et al., 2016), and encompassing both personal and societal values (Zimmerman et al., 2014). Resilience is also often viewed as an outcome, such as the absence of post-traumatic stress disorder (PTSD) or low levels of symptoms (Polousney et al., 2017). Resilience is also examined as a process, stemming from a systems-oriented perspective which views resilience as dynamic and flexible and expected to vary based on outcome of interest, over time, and across different contexts (Walsh, 2016a). This work often examines the development of resilience from a family systems (Walsh, 2016a, 2016b) and developmental (Masten, 2014; Sleijpen, June Ter Heide, Mooren, Boeije, & Kleber, 2013) perspective. Thus, it is critical to consider the ways in which various definitions of resilience shape the understanding of, and purported prevalence rates of, this construct. Although differing prevalence rates have been theoretically reasoned to be accounted for by differences in definitions, this has, to date, been theoretically addressed but not empirically tested in the same sample. There is a need for an examination of multiple definitions of resilience in a single, large, trauma-exposed sample.

Beyond definitional issues, nuances of sample and stressor are likely to influence the estimated prevalence of resilience. Resilience is often considered the rule rather than the exception (Bonanno, 2004; Kessler, Sonnega, Bromet, Hughes, & Nelson, 1995), and while this has been demonstrated in numerous military samples (e.g. Johnson, Polousney, & Erbes, 2011; Nash et al., 2015; Polousny et al., 2017), low levels of self-reported resilience have also been reported (e.g. Vyas et al., 2016). These conflicting findings may be consistent with another line of research suggesting that resilience is not necessarily the most common outcome, particularly following certain stressors (e.g. severe interpersonal traumas; Steenkamp, Dickstein, Salters-Pedneault, Hofmann, & Litz, 2012) or in certain populations, such as refugees (Sleijpen et al., 2013). Here, it has been suggested that it may be inappropriate to consider mental health problems as evidence of lack of resilience, with the presence of some psychological symptoms considered to be a normative reaction to extreme experiences or circumstances (Papadopoulos, 1999; Sleijpen et al., 2013; Southwick, Bonanno, Masten, Panter-Brick, & Yehuda, 2014). Varying definitions of resilience thus reflect different patterns of adaptive functioning, by different criteria. As noted by various resilience researchers, it is essential to clarify and measure resilience by a defined criterion of adaptation and the nature of adversity or stress (Luthar et al., 2000; Masten, 2015). In this study, we sought to illustrate and examine different patterns of resilience based on varying criteria, primarily via examination of prevalence rates, in a sample of individuals, all with exposure to combat trauma.

### 1.1. Comparing various models of resilience in the same sample

Comparing various resilience definitions within a single, well-characterized sample of individuals who have experienced a shared trauma type, i.e. military combat exposure, allows for greater empirical demonstration of this argument and examination of its impact. In the present work, we used data from an ongoing study to examine frequently used definitions of resilience as conceptualized as an outcome, as this represents the data available for this sample. Specifically, we focused on four definitions of resilience, referred to as models: Model 1: PTSD Model; Model 2: DSM-IV Psychopathology Model; Model 3: Generalized Distress Model; and Model 4: Trauma Load–Resilience Discrepancy Model. We then compared these models to an alternative conceptualization of resilience as a self-reported trait- or skills-based approach, e.g. the Connor–Davidson Resilience Scale (CD-RISC) (Connor & Davidson, 2003). Although we expected that different measures of resilience are likely to be related, the concordance may not be complete (e.g. one could have high perceived coping skills, suggesting high skills-based resilience, but still develop psychopathology following a traumatic event).

The overarching goal of this project was to directly examine and compare definitions of resilience in the same sample in order to extend, in an empirical manner, an ongoing theoretical perspective in the literature regarding the multidimensional nature of resilience and inconsistencies across diverse domains of adaptation, which complicates the delineation of resilience within individual studies (Luthar, Chicchetti, & Becker, 2000). To our knowledge, this has not been directly tested. More specifically, the comparison of these models is used to shed light on four questions: (1) What is the prevalence of resilience across models? (2) What is the concordance across models? (3) How related is self-reported trait-based resilience to outcome-based models? (4) Can one be classified as resilient but still have psychiatric distress? We address these questions through an empirical examination of different prevalence rates of resilience in a sample exposed to combat trauma, but based on varying resilience definitional criteria, to illustrate and further inform our understanding and conceptualization of resilience. We then discuss how the present findings may be viewed from the perspective of the systems-based approach to resilience and efforts to integrate across systems, and end with suggestions for useful directions for future research.

## 2. Study methods

### 2.1. Sample

The sample was selected from US military service members and veterans who participated in the Veterans Affairs (VA) Mid-Atlantic Mental Illness Research, Education and Clinical Center (MIRECC) multisite Post-Deployment Mental Health Study (PDMH Study) (Brancu et al., 2017). Inclusion criteria of the broader study entailed serving in the US military and/or reserve forces on or after 11 September 2001. Deployment or healthcare treatment-seeking were not requirements for study enrolment. Although advertised broadly, the sample is volunteer based and approximately half of the sample reported receiving outpatient mental health treatment in the past 3 years (either within and/or outside the VA system), although current treatment-seeking is unknown. For the current study, we examined a subset of this sample (*N* = 849) who reported military combat trauma exposure that met criteria for Diagnostic and Statistical Manual of Mental Disorders, 4th Edition (DSM-IV; APA, 2000) PTSD Criterion A1. Demographic characteristics of the sample are presented in Table 1. The rates of treatment-seeking and combat exposure requirement for inclusion in this analysis probably influenced the high levels of trauma exposure as well as the generally higher rates of PTSD and other psychiatric conditions in this sample compared to other military and veteran samples (Frayne et al., 2011; Hoge, Auchterlonie, & Milliken, 2006).

**Table 1.** Descriptive characteristics of the sample (*N* = 849).

	*N*	%
Gender		
Female	105	12.4
Male	744	87.6
Ethnicity ^a^		
White/Caucasian	456	53.7
Black/African American	362	42.6
Native American	26	3.1
Asian/Pacific Islander	16	1.9
Marital status		
Married	465	54.8
Never married	185	21.8
Divorced	137	16.1
Separated	60	7.1
Widowed	2	0.2
Employment status		
Employed full-time	418	49.2
Employed part-time	85	10.0
Not working/retired	343	40.4
Education		
General Equivalency Diploma (GED)	41	4.8
High school	346	40.8
Technical/trade school	89	10.5
Associate’s degree	149	17.6
Bachelor’s degree	129	15.2
Graduate degree	50	5.9
Other	44	5.2
Service era ^b^		
Operation Enduring Freedom	369	43.5
Operation Iraqi Freedom	726	85.5
Operation New Dawn	12	1.4
Gulf/Post-Gulf	522	61.5
Vietnam/Post-Vietnam	170	20.0
Branch of service ^b^		
Army	466	54.9
Army Reserve	126	14.8
Army National Guard	219	25.8
Navy	59	6.9
Navy Reserve	22	2.6
Air Force	28	3.3
Air Force Reserve	10	1.2
Air National Guard	8	1.0
Marines	107	12.6
Marine Reserves	14	1.6
Coast Guard	2	0.2

^a^ Data missing for three participants. ^b^ Some participants reported more than one category.

### 2.2. Procedures

Informed consent and data collection were completed at one of four VA medical centres; institutional review boards at each site approved the study. Following informed consent, participants completed both a diagnostic interview and a comprehensive self-report assessment battery within a 2 week period.

### 2.3. Measures and resilience models

Several interview and self-report measures were used to construct the models of resilience, as further described in Table 2. The Structured Clinical Interview for DSM-IV-TR Axis I Disorders: Patient Edition (SCID I/P, Version 2.0) (First, Spitzer, Gibbon, & Williams, 1994) was used for the determination of all current and lifetime mental health diagnoses, and for Model 1 (PTSD Model) and Model 2 (DSM-IV Psychopathology Model). The Global Severity Index (GSI) score was used from the Symptom Checklist-90 Item – Revised (SCL-90-R) (Derogatis, 1992) as a measure of generalized psychological distress. The GSI T-score was used for Model 3 (Generalized Distress Model). Model 4 (Trauma Load–Resilience Discrepancy Model) used the GSI as well as the Traumatic Life Events Questionnaire (TLEQ) (Kubany et al., 2000). The TLEQ was used to assess exposure and response to 23 listed traumatic events, in those with at least one military combat trauma exposure. Trauma load was defined as the number of Criterion A1 potentially traumatic event categories endorsed one or more times, in line with previous work (e.g. Kolassa, Kolassa, Ertl, Papassotiropoulos, & De Quervain, 2010). As used in prior analyses in other samples (e.g. Amstadter, Myers, & Kendler, 2014; Sheerin et al., 2018), Model 4 defined resilience as the difference between actual and predicted GSI scores given the number of lifetime traumatic exposures. GSI was the outcome variable and TLEQ was the predictor variable; the TLEQ scores for pre-, during, and post-military trauma occurrences were entered hierarchically into the linear regression model to control for the effect of pre-military trauma exposure on current psychological symptoms. The GSI and trauma load data met the assumption of linearity, but demonstrated considerable heteroscedasticity. The weight estimation procedure was employed, which is preferred when there are differences in variability of a given variable (i.e. GSI scores) across the range of possible values (i.e. TLEQ trauma count). GSI T-score was specified as the weighted variable in the equation. Standardized residuals from the linear regression were then saved, dichotomized, and multiplied by one for ease of interpretation, to create the resilience categories (i.e. resilient cases, positive residuals, representing lower than expected distress given trauma load; and non-resilient cases, negative residuals, representing higher than expected distress). Finally, the total score on the CD-RISC (Connor & Davidson, 2003) was used to compare and contrast self-reported resilience as a function of resilience status across the four models.

**Table 2.** Resilience model definitions and prevalence rates in the study sample.

Model	Definition/determination	Prevalence of resilience
Model 1^a^: PTSD Model	Categorized individuals based on the presence or absence of current diagnosis of PTSD or sub-threshold PTSD	43.7% (*n *= 371)
Model 2^b^: DSM-IV Psychopathology Model	Categorized individuals based on the presence or absence of any current (Axis I) DSM-IV disorder	30.7% (*n *= 261)
Model 3^c^: Generalized Distress Model	Caseness was operationalized as having a T-score ≥ 63 on the GSI of the SCL-90-R	87.4% (*n *= 742)
Model 4^d^: Trauma Load–Resilience Discrepancy Model	Defined as the difference between actual and predicted GSI scores given number of lifetime traumatic exposures as measured by the TLEQ. Standardized residuals from the linear regression model were then dichotomized for purposes of classifying resilient (lower than expected distress given trauma load) and non-resilient cases (higher than expected distress)	51.1% (*n *= 434)

^a^ Bonanno, Galea, Bucciarelli, & Vlahov (2006); ^b^ North et al. (2002); ^c^ Galatzer-Levy, Burton, and Bonanno (2012); ^d^Amstadter et al. (2014).

PTSD, post-traumatic stress disorder; DSM-IV, Diagnostic and Statistical Manual of Mental Disorders, 4th Edition; GSI, General Severity Index; SCL-90-R, Symptom Checklist-90 Item – Revised; TLEQ, Traumatic Life Events Questionnaire.

## 3. Results

### 3.1. Prevalence across models

Prevalence rates of resilience by model (Table 2) are as follows. Model 1: 43.7%; Model 2: 30.7%; Model 3: 87.4%; Model 4: 51.1%. (Note that, as a function of the analytic approach (deriving resilience using residuals from a regression), prevalence estimates based on presence/absence will, by definition, be close to half.) Thus, resilience was most common with a criterion of general distress (Model 3) and least common when using a stringent definition of any current DSM-IV psychiatric diagnosis.

### 3.2. Concordance across models

The kappa statistic was used to determine the concordance and strength of association regarding the number of individuals considered resilient when comparing models. The concordance between Models 1 and 2 was statistically significant (36.2% of the sample with both PTSD and another psychiatric disorder; kappa = .10, *p* < .001; however, this should be interpreted with caution given PTSD in both models), as was the concordance of resilient cases in Model 3 by Models 1 and 2 (30.5% of the total sample considered resilient by all three models, kappa = .11, *p* < .001). Finally, adding in Model 4, the number of individuals who could be considered resilient by all four models was 25.7% (*n* = 218) (kappa = .40, *p* < .001).

### 3.3. Comparing trait-based and outcome-based models

We then examined the resilience models in comparison to CD-RISC scores (Table 3). The *t*-tests showed that mean CD-RISC scores were higher among those classified as resilient by all four models. CD-RISC scores were highest for those classified as resilient by Model 2 and lowest by those classified as non-resilient by Model 3. Correlations between resilience models and CD-RISC scores were moderate.

**Table 3.** Results of *t*-tests examining differences in Connor–Davidson Resilience Scale scores by resilience outcome model.

Model	*M*	*SD*	*t*	df	*p*	CI	*r*
Model 1			14.1	846	< .001	13.8–18.3	0.43
Resilient	77.8	16.2					
Non-resilient	61.7	16.9					
Model 2			13.6	846	< .001	14.5–19.3	0.47
Resilient	81.3	13.1					
Non-resilient	64.4	18.0					
Model 3			10.8	846	< .001	15.7–22.7	0.46
Resilient	72.1	16.7					
Non-resilient	52.8	20.4					
Model 4			17.1	846	< .001	16.5–20.8	0.51
Resilient	78.7	14.1					
Non-resilient	60.1	17.5					

Model 1, ± post-traumatic stress disorder; Model 2, ± DSM-IV Psychopathology; Model 3, above/below cut-off on Global Severity Index, Generalized Distress Model 4, ± standardized residual for Trauma Load–Resilience Discrepancy Model.

### 3.4. Resilience with psychiatric distress

We further explored the relationships between the self-reported measures of resilience (i.e. CD-RISC), the observer-based clinical diagnoses of Model 2, and the trauma load–resilience discrepancy of Model 4. Among those in the 75th percentile of resilience scores on the CD-RISC (i.e. those with the highest self-reported resilience coping skills), 42.3% had a current DSM-IV disorder (i.e. considered non-resilient by Model 2). Thus, a substantial number of individuals who demonstrate the highest levels of resilience coping skills may also endorse sufficient distress to warrant a psychiatric diagnosis. When high CD-RISC was examined in the context of Model 4, 86% were in the resilient group. Further examination of Model 4 by resilience status and by examination of quartiles within this model is presented in Table 4. Many individuals classified as resilient were also diagnosed with a DSM-IV disorder, indicating that some individuals may report lower than expected generalized psychological distress given trauma load and yet meet criteria for a psychiatric diagnosis. Examination of quartiles suggests that although the percentage of individuals with DSM-IV psychopathology decreased in subsequent quartiles, individuals at the highest levels of resilience, as defined by the trauma load–resilience discrepancy model, may still experience significant psychiatric symptoms.

**Table 4.** Prevalence of psychopathology as a function of resilience status and quartiles in the Trauma Load–Resilience Discrepancy Model.

	Presence of DSM-IV Axis I psychopathology
Non-resilient (*n* = 415)	89.6% (*n* = 371)
Resilient (*n* = 434)	49.8% (*n* = 216)
Quartiles from low to high resilience	
1st quartile	71% (*n* = 77)
2nd quartile	23.6% (*n* = 51)
3rd quartile	22.7% (*n* = 49)
4th quartile	18.1% (*n* = 39)

## 4. Discussion

### 4.1. What is the prevalence of resilience across models?

Our examination demonstrated that the number of individuals who were classified as resilient varied widely across models (30.7–87.4%). In agreement with some extant findings (e.g. Infurna & Luthar, 2016a), with the exception of the broader generalized distress model (Model 3), resilience, as defined by these specific outcomes, was not the most common response in this sample. This is likely to be due in part to specific characteristics of this sample (e.g. combat trauma exposure inclusion criteria, high rate of past mental health treatment-seeking) and strict requirements for some of the resilience determinations (i.e. presence of any psychopathology was deemed non-resilient). Indeed, rates of PTSD and other DSM-IV disorders in our sample are higher than in other military and veteran samples (e.g. Frayne et al., 2011; Hoge et al., 2006); as such, it is unknown how representative this sample is compared to other trauma-exposed samples. However, regardless of the specific prevalence rate of resilience in this sample, our aim was to highlight the varying rate of resilience across different definitions in the same sample.

### 4.2. What is the concordance across models?

While there was noted concordance across many models, these rates were modest and, not surprisingly, our examination demonstrated a notably smaller proportion of individuals considered resilient by all models. The relatively modest concordance rates within the same sample are in line with recent work by Infurna and Luthar (2016a), albeit with a single time-point outcome. This reflects the important differences in classifications of resilience and further supports their suggestion that any estimation of rate should be interpreted with caution and with awareness of the approach used to define resilience (Infurna & Luthar, 2016b).

### 4.3. How related is self-reported trait-based resilience to outcome-based models?

The finding of strong associations, but not unity, between outcome-based models and the CD-RISC further supports resilience as encompassing both outcomes and skills/traits. This suggests that these types of measures are capturing different facets of resilience. The dynamic systems perspective of resilience notes the importance of integrating resilience within and across different systems (Walsh, 2016a, 2016b). In line with this perspective, the present findings highlight the potential usefulness of incorporating different measures (e.g. within the individual system), measuring various outcome-based and coping-based resilience perspectives to arrive at a more complete understanding of an individual’s response to adversity.

### 4.4. Can one be classified as resilient but still have psychiatric distress?

Many individuals deemed to be resilient by the trauma load–resilience discrepancy model and/or by high CD-RISC scores also had DSM-IV diagnoses. This is likely, in part, to be due to the sample. For example, in the context of military culture, personnel are taught and expected to continue their tasks and respond to orders without complaint. It may also be a nature of the sample selection itself, which was highly trauma exposed, and approximately half of the sample had sought mental health treatment in the past. This finding is, however,consistent with one perspective of resilience that encompasses the possibility that individuals can be resilient and yet still be suffering from negative outcomes of the trauma (Sleijpen et al., 2013; Southwick et al., 2014). These findings also align well with the systems perspective that would expect variability in resilient responses, as a function of outcome of interest, context, and impact of other systems (e.g. community, family, biological) (Walsh, 2016a).

### 4.5. Suggestions for future directions

Using varying outcome-based definitions of resilience within the same sample, the present study findings highlight important conceptual issues for the resilience literature. Here, we offer some suggestions for continued resilience research to better conceptualize and understand this multifaceted construct.

First, the examination of continuous measures of outcomes is important given that resilience is a complex construct not easily distinguished into clear categories and there is wide variation in adaptation following adverse events (Luthar et al., 2000). The residuals methodology was dichotomized in the present analyses to compare to a commonly used resilience outcome determination. However, it is noted that dichotomizing the residuals will necessarily result in approximately 50% in either group; this methodology is probably better suited for use as a continuous measure, and is currently being used in this manner in ongoing work in our laboratory. Using the residuals as a continuous measure allows for examination of the degree of variance around the imposed mean. As has been insightfully pointed out in their commentary of this methodology, Wertz and Pariante (2014) noted that individuals can be considered resilient with varying differences between their actual and expected symptoms; there may be qualitative differences between those with small differences and those who are doing far better than expected. Further examination of this variability will be useful in better describing and understanding resilient individuals.

Secondly, the comparison of outcome-based resilience models with the self-reported CD-RISC supports the operational definition of resilience as a set of coping skills and traits, as well as positive adaptation and outcomes following trauma exposure. It has been argued that resilience should be domain based (Infurna & Luthar, 2016a), with domains clearly delineated, and that multiple domains/outcomes should be examined across varying systems. In this analysis, we compared three commonly used definitions of resilience: diagnosis (both presence/absence of PTSD and presence/absence of broad psychopathology), psychiatric distress, and perceived coping ability. Other domains, such as quality of life, substance abuse, and role functioning, should also be considered in combination, as examining only one domain of resilience is incomplete (Infurna & Luthar, 2016b). For that reason, stating a percentage of resilience in our sample based on any one of our definitions is not appropriate. Despite the limitations of a single definition of resilience, the literature as a whole continues to examine and quantify resilience using a single definition, most commonly the presence/absence of PTSD symptoms (e.g. Bryant et al., 2015; Greene et al., 2017; Polousny et al., 2017). While useful, given that trauma is a transdiagnostic factor, this approach does not capture other, important patterns of responding. Examination of resilience from multiple perspectives, as compared to absence of PTSD symptoms (e.g. Bonanno, Galea, Bucciarelli, & Vlahov, 2006; Greene et al., 2017; Polousny et al., 2017) or high self-reported resilience coping (e.g. Green, Beckham, Youssef, & Elbogen, 2014; Pietrzak et al., 2010) in isolation, will be important for practitioners and the military for purposes of prevention and intervention programming, as well as assessment of redeployment readiness.

As Masten (2015) has argued, resilience is dynamic, as individuals and their environmental context are always changing; thus, whether defined by capacity, outcomes, or processes of positive adaption following risk, resilience will depend on the co-action of multiple systems interacting in the functioning of an individual. Future research should incorporate multiple domains of functioning within and across systems, examine these domains and processes over time, and further explore the disconnect between different domains and definitions if and when this occurs. For example, in a military sample, this would include the recognition that with regard to context associated with military trauma, this includes unit support (linked closely in time to trauma exposure) as well as the shift in context from military to post-deployment experiences and reintegration into the community and with the family. Researchers must be aware of the changing context over time (i.e. dynamic systems) and the relevance to resilience and subsequent outcomes. From an empirical standpoint, this would include examination of time-variant covariates in longitudinal, trajectory analyses.

A third point of discussion is associated with findings that resilience by some definitions is not necessarily equal to being symptom free. This adds to the ongoing debate over different approaches to resilience: those that capture stable functioning or those that can include ongoing symptoms and distress (see discussion in Southwick et al., 2014). This perspective may be particularly relevant in highly trauma-exposed populations and may be considered in the context of Hobfoll, Stevens, & Zalta (2015) discussion of ‘resistance to breakdown’, described as a specific attribute of resilience, wherein individuals may be relatively unharmed up to a point, but then experience an event that renders them no longer able to respond positively (i.e. the straw that breaks the camel’s back effect). It is also worth clarifying that resilience in some domains does not imply positive adaptation across all functional domains and outcomes (Luthar et al., 2000; Sleijpen et al., 2013). Capturing these different definitions of resilience, although complicated, will ultimately result in a much richer understanding of the wide variation in responding following trauma and stressful life events. Longitudinal designs, and those using a systems-based perspective (Masten, 2016), are needed to better capture the dynamic process of resilient responding across definitions. While it may be prudent in the future to specifically categorize and name different types of resilience, at present it is suggested that researchers be clear about their definitions, and specify the particular areas to which their data apply (Luthar et al., 2000), as this directly impacts the conclusions and implications.

### 4.6. Limitations

In our sample, we attempted to limit heterogeneity by requiring exposure to a combat trauma in order to be included in the analytic sample. However, there remain differences in time since trauma, the inability to capture the severity or intensity of traumatic events, and differences in pre- and post-military trauma history. Measuring these trauma characteristics will be important in future research. As the sample was cross-sectional, our analysis did not take into account the concept of ‘relative resistance’ (see commentary by Hobfoll, Stevens, & Zalta, 2015), in that we are not able to separate out current symptoms and distress from pre-existing, pre-trauma levels of distress and functioning, nor were we able to examine causality. Longitudinal investigations designed to separate pre-existing, chronic, and new-onset conditions will be informative. Longitudinal and trajectory-based examinations of resilience (e.g. Andersen, Karstoft, Bertelsen, & Madsen, 2014; Fink et al., 2017; Norris, Tracy, & Galea, 2009) that measure the timing of traumatic events and dates of onset for diagnostic conditions are essential. The examination of new-onset or ongoing stressors in these studies is suggested to provide a more nuanced picture of the process of resilience in the face of ongoing adversity to better understand the relationships among resilience antecedents, attributes, consequences, and long-term trajectories of resilience and stress. Another limitation is that given the nature of the study, we focused on resilience definitions based on outcome. Thus, findings are limited with regard to their ability to extend to other approaches, particularly process-oriented, systems-based approaches. Finally, the volunteer nature of the sample of primarily treatment-seeking participants is a limitation, in that results are probably not as generalizable to Afghanistan and Iraq-era veterans as a whole, or as generalizable they would be if random sampling was utilized. This sample is, however, similar to other existing studies of treatment-seeking military personnel (Vyas et al., 2016) and is representative of veterans likely to be presenting for treatment.

### 4.7. Conclusions

The field’s understanding of resilience has been challenged by the definitional difficulties inherent in the construct, and there is wide variation in the individual’s post-stressor behaviour or health domain of functioning that is measured. Comparing and contrasting different definitions of resilience among a sample exposed to combat trauma from a data-driven framework empirically demonstrated these varying rates as well as the benefits of combining multiple measures of resilience in the same sample (e.g. outcome based and coping based) to gain a more nuanced understanding of resilient responding. The findings demonstrated that resilience classification varied across definitions, the majority of individuals across models were not categorized as resilient, and many categorized as resilient in the trauma load–resilience discrepancy model, as well as those endorsing high levels of skills-based coping and perceived resilience, also had current DSM-IV-diagnosed conditions.

Our illustration of the variability in resilience categorizations adds support to the idea that the prevalence of resilience is dependent upon the definition employed. As such, determination and description of resilience should not rely on a single domain. It is being increasingly recognized that competence in one domain does not preclude difficulties in other domains of functioning (Luthar, Doernberger, & Zigler, 1993; Masten, 2014; Southwick et al., 2014). It has also been suggested that resilience should be further separated into different facets (Hobfoll et al., 2015). Our findings support the benefits of systems-oriented approaches which view resilience as dynamic and expected to vary based on outcome of interest, over time, and across different contexts. There have been calls to increase efforts to integrate findings and their applications within and across system levels (Borge, Motti-Stefanidi, & Masten, 2016; Masten, 2016) in order to best capture and understand the multifaceted nature of resilience. As the field progresses, examination of multiple domains of resilience and ongoing changes in this response (over time and in response to new, ongoing stressors) will undoubtedly shed new light on our understanding of this multifaceted construct.
